# Integrating MICRORNA941 and T cell subset research into public health strategies for managing inflammaging in elderly and immune-compromised patients

**DOI:** 10.6026/9732063002001529

**Published:** 2024-11-30

**Authors:** Lily Fotovat, Kathleen Wang, Francesco Chiappelli

**Affiliations:** 1 Dental Group of Sherman Oaks, Sherman Oaks, CA - 91403, USA; 2 UCLA Center for the Health Sciences, Los Angeles, CA - 90095, USA

**Keywords:** Immunosenescence, inflammaging, MicroRNA941, senescence-associated secretory phenotype (SASP), infectious diseases (CoViD-19, PACS Long Covid)

## Abstract

As of 2022, the Centers for Disease Control and Prevention (CDC) reported that the average life expectancy for both sexes in the United States is 77.5 years. While new advances in health have increased life expectancy, aging comes with complications that impact the development of diseases like cancer, senile dementia (non-Alzheimer), diabetes and Parkinson's. Through aging, the body's immune system declines, a process recognized as immunosenescence and which contributes to inflammaging, a state of chronic, though non-productive, inflammation that progresses with advancing age in the absence of overt infection and that contributes to the onset and progression of a spectrum of age-related pathologies. MicroRNAs are small forms of RNA that control gene expression by binding to messenger RNA (mRNA) in the cell cytoplasm. In particular, microRNA-941 (miR-941) has been found to play a critical role in the regulation of differentiation of cell populations, certain T cell subsets responsible for maintaining efficient immune surveillance in normal subjects, immune compromised individuals as well as the elderly. We propose that concerted research designed to define and characterize interventions targeting the regulatory effects of miRNA-941 specifically on T-cell subsets will benefit treatment of infectious (*e.g.*, CoViD-19, H5N1 infection) and chronic illnesses (*e.g.*, diabetes II, diabetes III, Long Covid [*i.e.*, Post-Acute Covid-19 Syndrome, PACS], autoimmune disease), which are most common among the aging and the immune compromised population. It is possible and even probable that active research in this specific area will proffer new horizons for finding cures, aid in disease management and improved accessibility and affordability of public health services.

## Background:

As of 2022, the Centers for Disease Control and Prevention (CDC) reported that the average life expectancy for both sexes in the United States is 77.5 years [1]. While new advances in health have increased life expectancy, aging comes with complications that impact the development of diseases like cancer, senile dementia (non-Alzheimer), diabetes and Parkinson's [2]. Through aging, the body's immune system declines, a process accompanied by, responsible for and possibly engendered by inflammaging. In addition, the steady increase in life expectancy impacts people's daily lives and public healthcare. With people living longer, finding affordable, reliable and alternative treatments for age-related diseases is vital to avoid placing further strain on healthcare systems [2]. As people get older and live longer, new technologies and interventions must be developed to help combat the diseases that arise with old age and promote the concept of healthy aging while considering environmental and geographical factors [2].

Therefore, it is urgent to find solutions to combat immunosuppression and inflammaging. MicroRNAs can recognize cancer and develop disease biomarkers [3]. Additionally, since the function of certain T-cell subsets decrease with age, studying T-cells that respond to the CD28 co-stimulus by activation and proliferation can offer insights into the behavior and survival of this and other T-cell subsets [4]. Emerging evidence suggests that looking at microRNA and T-cell interventions and research can help inform future public health strategies for managing inflammation in the elderly.

## Understanding inflammaging in the elderly:

Biologically, aging refers to the physiological process of tissue degeneration related to chronic inflammation. This age-related chronic inflammation is highly associated with inflammaging, an imbalanced immune system that can develop through aging or external triggers [2]. Immune cells, vital for identifying and clearing these senescent cells, are impacted by senescence-associated secretory phenotype (SASP), contributing to the decline in immune function known as immunosenescence [2]. Immunosenescence, a process associated with aging that impairs the immune function, is responsible for inflammaging [2].

The consequences of both mechanisms may lead to increased susceptibility to infection and poor responses to vaccination, as well as to chronic inflammatory and degenerative diseases in the elderly [5]. Moreover, the accumulation of senescent cells can trigger inflammation in organs, leading to organ damage and an increased risk of age-related diseases like cancer [6]. This process is heightened by positive feedback loops that promote the build-up of inflammation and organ damage, amplifying inflammation and increasing the risk of age-related diseases [6]. As people grow older, more persistent illnesses, including Alzheimer's disease (now sometimes referred to as Type III diabetes) and cancer, significantly affect people's quality of life. People with high socioeconomic status (SES) can afford treatment or newer technologies to combat diseases that affect them, while those with lower SES cannot: the higher the SES, the higher the life expectancy, as disparity in SES becomes an issue of resource access [7]. A key result is that in older age, those with the most significant health needs are often the ones with the least access to resources to manage their care [7].

## MicroRNA941:

MicroRNAs are a class of small noncoding RNAs which function in post-transcriptional regulation of gene expression [8]. They are powerful regulators of various cellular activities, including cell growth, differentiation, development and apoptosis [8]. MicroRNA expression profiling is used to classify several different human cancers and can also distinguish malignant tumors from benign tumors [9]. MicroRNA-941 (miR-941) is found in high amounts in cells that can potentially become any type of cell (pluripotent cells). Still, its levels drop when these cells begin the process of differentiated specialization [9]. In humans, miR-941 affects brain cells and influences genes involved in neurotransmitter signaling, which helps control how brain cells communicate. The observation that miR-941 is associated with certain cancers (*e.g.*, breast, liver, lung, laryngeal), cardiovascular inflammation and certain autoimmune diseases (*e.g.*, psoriatic arthritis) suggests that it also plays a critical role in regulating cellular immunity. Taken together, these lines of evidence confirm that miR-941 is a key biological regulator in human cell development, immune surveillance and brain function [9].

To be clear, miR-941 inhibitors - *i.e.*, chemically-modified oligonucleotides that hybridize with mature miRNAs - can significantly lower miR-941 levels in breast cancer cells, depending on the dose used [9]. Data show that miR-941 regulates cancer cell growth by modulating histone phosphorylation and consequentially gene expression. MiR-941 inhibitor showed strong anticancer effects in lab-grown cancer cell growth and proliferation in vitro [9]. In other studies, miRNA-pathway network analysis showed that miR-941 can regulate T-cell receptor signaling, insulin signaling and MAPK signaling. MiR-941 may act as a reliable biomarker of Acute Coronary Syndrome and could predict the severity and progression of coronary heart disease [3, 10]. While miR-941 may have evolved to enhance human longevity by maintaining stem cells, it may also inadvertently increase cancer risk, highlighting a trade-off between longer life and vulnerability to disease [10]. This dichotomy further suggests that miR-941 may support longevity but at the cost of making cells more susceptible to cancerous changes [10]. Additional studies have shown that the pattern of miRNA expression is a reliable tool for predicting therapeutic outcomes in cancers such as leukemia and colon cancer. Levels of miR-143 and miR-145, related to miR-941, are often lower in many types of cancer [11]. When researchers restored these miRNAs in prostate or colorectal tumors, they saw a significant reduction in tumor growth in lab-grown cells and living organisms [11]. Additionally, the role of miRNAs can vary depending on the type of cell they are in. For example, miR-29a can act either as a cancer-promoting gene (oncogene) or as a cancer-suppressing gene (tumor suppressor), depending on the specific environment within the cell [11].

## T cell subset research:

T-cells, otherwise known as T-lymphocytes, are highly specialized immune system cells. Like many other specialized cells, T-cells develop from stem cells in the bone marrow and finalize their maturation as thymocytes [12]. T-cells are responsible for cellular immunity and produce and regulate the cytokines that mediate inflammation. Cytokines are signaling proteins that trigger immune system activity to control cell growth and maintain the body's immune and inflammatory responses [13]. Previous studies have uncovered an array of differentiated CD4+ T-cells such as T helper 1 (TH1), TH2, TH17 and T follicular helper (Tfh). These cell subsets possess developmental and regulatory pathways that play critical roles in immunity [13]. In addition, the proportion of naïve T-cells in the CD4+ and CD8+ T-cell subsets sharply decreases with aging, particularly for the CD8+ population [14]. During aging, the thymus, the organ responsible for generating self-tolerant and immune-competent T-cells, undergoes thymic involution [15]. When this occurs, new naïve T-cell production decreases. T-cell subset reservoir severely decreases with age. The functional response of T cells may also decrease in aging, in addition to their numbers. Together, the drop in T cell number and function may severely impact immune function in the aging population. A decrease in the generation of functional T-cells with advancing age in humans may significantly contribute to the onset of several types of cancer and chronic or infectious diseases [15].

One mechanism that may bring about aging-associated loss of T cell activity may be due to their decreased response to co-stimulation by the 90KDa constitutive membrane-associated glycoprotein referred to as cluster of differentiation #28 (CD28), an essential co-stimulatory molecule for T cell activation and proliferation [15]. With aging, CD28 response decreases, as consequentially does overall reduced immune surveillance and, specifically with respect to current threats of infectious diseases including SARS-CoV2 and influenza H5N1, decreased response to vaccinations [4]. In addition, CD4+ T-cells play a role in the pathogenesis of rheumatoid arthritis (RA), an autoimmune disease characterized by joint pain, stiffness and swelling [16]. Studies observed that RA patients have an increased frequency of aged T-cells. Chronically activated T-cells derived from inflamed joints may be due to prolonged exposure to pro-inflammatory cytokines such as tumor necrosis factor (TNF)-α [15, 16]. Observations of a model of T-cell activation in chronic inflammation predicted that the environment of inflamed joints is significantly different from that of a small inflammatory episode. Chronic exposure of T-cells to TNF uncouples T-cell receptor (TCR) signaling pathways and thus impairs T-cell subset functionality [15, 16]. CD28-memory T-cell subsets contribute to inflammation and may also aggravates the disease severity of RA upon stimulation by increasing the production of inflammatory factors [15, 17]. Indeed, a reduction of CD8+ CD28-T-cells in RA patients was distributed and favorable clinical responses resulted [17].

Memory T-cells in aging individuals exhibit the pro-inflammatory phenotype SASP, which causes chronic inflammation [15, 18]. These memory-like T-cells show diminished expression and response to co-stimulatory molecules, including CD28, which regulate T-cell activation and preventative functions within the body's immune system [15, 18]. Without the continual support of such co-stimulatory molecules, the memory T-cells show decreased telomerase activity and increased DNA damage [15, 18]. The age-dependent preferential accumulation of memory T-cells contributes to inflammaging and tissue deterioration. By contrast to miR-941, another micro-RNA form (*i.e.*, miR-155) is required to accumulate active CD4+ T and Tfh cells [11]. Similarly, miR-146a blunts severe inflammation by decreasing the activation of T-cells [15, 19]. Nonetheless, miR-146a-/- (knockout) mice undergo a significant life expectancy decrease due to extreme immune dysfunction and chronic inflammation caused by the early activation of CD4+ T-cells [20]. Additionally, when miR-155 is expressed and miR-146a is not expressed, miR-155 is responsible for the accumulation of CD4+ T and Tfh cells, the spontaneous formation of B cells within the germinal centers and cell-attacking autoantibodies. Thus, because miR-155 initiates age-dependent pro-inflammatory responses in T-cells, it plays a crucial role in inflammaging in the murine model [20, 21]. The implications of these findings in treating inflammaging in humans remain to be tested.

## Future strategies:

As a society, aging and having a longer life span should be viewed in a good light as it allows young people to plan their lives with greater security [7]. Healthcare needs to address the multidimensional demands of older age in an integrated way instead of primarily focusing on services that treat disease independently to meet the needs of older adults and impose significant costs on them and the health system [7]. Further research should be conducted, like comparing blood tests across four test groups in elderly populations, all in the same nursing home: cancer, senile dementia (non-Alzheimer's), diabetes II and Parkinson's disease. A recent study tested how biomarkers might differentiate between various health conditions in an elderly population [22]. The study compared two groups of individuals aged 70 to 82: one group with chronic illnesses from significant cardiovascular, neurodegenerative and chronic pulmonary diseases, diabetes II and cancers (referred to as non-healthy aging, NHA). The control group consisted of age-matched individuals in good health (referred to as healthy aging, HA). The study performed quantitative analysis on forty pro-inflammatory cytokines in blood plasma and over 500 proteins in urine to identify and establish significant differences in potential biomarkers and understand the biological pathways associated with healthy aging [22]. Similar study designs in further research could refine the approach to monitoring and managing chronic diseases in aging individuals.

## Conclusion:

Since people are living longer, investing in technologies and a healthcare infrastructure that supports the influx of people aging is essential. Aging involves physiological tissue degeneration driven by chronic inflammation, linked to an imbalanced immune response known as inflammaging. This process leads to immunosenescence and increases vulnerability to infections and age-related diseases [2]. These effects are further intensified by socioeconomic disparities that limit access to essential healthcare resources for those most in need. Understanding and targeting the pathways of T-cells and microRNAs should lead to new and improved strategies and therapies to alleviate inflammaging and chronic diseases. For example, research should test novel modes of implementation and integration of microRNAs to control and regulate the expression of CD28 and other co-stimulator proteins [4]. In addition, ensuring gene expression of miR-146a, miR-155, miR-941 and other anti-inflammatory microRNAs should tested for improving treatment interventions of auto-immunity, chronic diseases and immune deficiencies [20]. Case in point, exploring treatments in miRNA-941 and T-cell subset research can be beneficial to understanding and treating aging-associated chronic illnesses. Researching these areas at an evidence-based transactional public health level will open up new possibilities for finding cures, aiding in disease management and hopefully improving accessibility and affordability. Such progress could improve the quality of life, reduce healthcare costs and enhance community health and resilience.
